# A modified TALEN-based system for robust generation of knock-out human pluripotent stem cell lines and disease models

**DOI:** 10.1186/1471-2164-14-773

**Published:** 2013-11-09

**Authors:** Stefan Frank, Boris V Skryabin, Boris Greber

**Affiliations:** 1 Chemical Genomics Centre of the Max Planck Society, Dortmund, Germany; 2 Human Stem Cell Pluripotency Group, Max Planck Institute for Molecular Biomedicine, Münster, Germany; 3 Institute of Experimental Pathology (ZMBE), University of Münster, 48149 Münster, Germany; 4 Interdisciplinary Centre for Clinical Research (IZKF), University of Münster, 48149 Münster, Germany

**Keywords:** Human pluripotent stem cells, Targeted gene disruption, TALE nucleases, Disease modeling

## Abstract

**Background:**

Transcription activator-like effector nucleases (TALENs) have emerged as a tool for enabling targeted gene editing and disruption in difficult systems, such as human pluripotent stem cells (hPSCs). The modular architecture of TAL effectors theoretically enables targeting of any genomic locus and several cloning systems for custom TALEN assembly have recently been established. However, there is a lack of versatile TALEN expression systems applicable to hPSCs.

**Results:**

Here, we extend an existing TALE assembly system by a dual set of expression vectors for efficient application of TALEN technology in hPSCs. This is characterized by improved TALEN architecture as well as antibiotic resistance and fluorescent reporter cassettes, thus enabling enrichment for transfected cells.

Improved functionality of the combined system was demonstrated by targeted disruption of the *HPRT1* gene to create isogenic disease models of Lesch-Nyhan-Syndrome. Using female hPSCs, homozygous disruption of *HPRT1* occurred at efficiencies of up to 15%. Differentiating isogenic knock-out cells both into central nervous system (CNS) as well as into sensory-like neurons recapitulated previously described phenotypes based on patient-specific induced PSCs and extended these findings to non-CNS neurons, respectively.

**Conclusion:**

The combined vector system allows for flexible and affordable generation of knock-out hPSCs lines, thus enabling investigation of developmental processes as well as the generation of isogenic disease models without the need for patient material.

## Background

Gene targeting in human pluripotent stem cells (hPSCs) by conventional approaches is a cumbersome and inefficient process. The development of sequence-specific nucleases, such as TALENs, can, however, significantly enhance the efficiency of genome editing in hPSCs [1]. TALENs are chimeric fusions between custom-designed transcription activator-like effectors (TALE) of *Xanthomonas* plant pathogens and the FokI nuclease [2-4]. Within the TALE structure, individual repeat domains confer specific recognition and binding to single nucleotides on DNA. Several types of repeat domains differing solely in their so-called repeat variable di-residues (RVDs) have been found to be selective binders of individual DNA bases, with differing affinities [5]. Custom design of the modular TALE repeat domain structure hence allows specific targeting and binding of TALEs to genomic regions of interest. Upon presence and adjacent binding of two TALENs, a DNA double-strand break (DSB) will be induced by the fused catalytic domain of FokI nuclease, which is then repaired either by the error-prone mechanism of non-homologous end joining (NHEJ) or via homology-directed repair [6]. Hence, in the absence of homologous template sequence, small genetic lesions may be introduced into a predefined locus by delivery of pairs of specifically designed TALEN constructs into cells, such as hPSCs [1,7].

Several approaches have been used for the generation of custom TALENs [7-11]. These are, however, not easy to adopt for new researchers entering the field [12]. Cermak *et al.*[13] have recently established a particularly straightforward TALEN assembly system that is based on GoldenGate cloning. It enables reliable TALEN assembly within a few days and has been made available through the Addgene repository. However, the associated expression vectors were not optimized for applications in mammalian cells. We have therefore developed a new set of vectors compatible with this publicly available TALEN assembly kit [13]. Our plasmids contain an improved TALEN backbone architecture incorporating findings by Miller *et al.*[6] as well as selection cassettes to enrich for transfected cells. Here, we report the application of the combined system to hPSCs by creating isogenic knock-out models for the X-linked *HPRT1* locus at high efficiencies. Mutations in this gene cause Lesch-Nyhan-Syndrome (LNS), a disease with strong neurological symptoms [14,15]. Clonal knock-out lines showed impaired differentiation into different neuronal lineages, recapitulating aspects of the disease phenotype *in vitro*. The combined TALEN assembly-and-expression system simplifies the custom generation of hPSC knock-out cell lines and will therefore be universally applicable for functional studies and the generation of hPSC-based disease models.

## Methods

### Cell culture

hPSCs were cultivated on Matrigel® in either MEF-conditioned or defined FTDA media [16,17]. HEK-293 T cells were cultured on conventional tissue culture plastic in DMEM supplemented with 10% fetal bovine serum, non-essential amino acids, 2-mercaptoethanol, and Penicillin-Streptomycin-L-Glutamine.

### TALEN design and assembly

TALE repeat structures were designed using either the ZiFit targeter http://zifit.partners.org/ZiFiT/) or the TAL Effector Nucleotide Targeter 2.0 (https://tale-nt.cac.cornell.edu/node/add/talen). TALENs were assembled as published [13], by following the protocol associated with the GoldenGate TALEN and TAL Effector Kit 1.0 (Addgene #1000000016), except that TALE repeats were ultimately cloned into vectors pTAL7A (for TALEN A) and pTAL7B (for TALEN B) (Additional file 1: Figure S1).

### Pretesting of TALENs and generation of HPRT1 knock-out lines

TALENs A and B were transfected into HEK-293 T cells or hPSCs at equimolar ratios using Fugene 6 (Roche). Starting from 24 hours after selection, antibiotic selection was applied for 48 hours with puromycin at 0.5 μg/ml and blasticidin at 5 μg/ml. After selection, cells were dissociated and (i) lysed to purify genomic DNA, (ii) analyzed via flow cytometry, or (iii) reseeded for selection of knock-out cells using 6-thioguanine (6-TG, Sigma, #A4862). Mutation frequencies were determined with the CEL-1 assay (Surveyor Nuclease S, Transgenomic, #706020) according to the manufacturer’s protocol. 6-TG was applied for 4–8 days at a concentration of 30 μM.

### Neuronal differentiation

Differentiation of hPSCs into neurons was performed as previously described [18,19]. Quantification of neurite length and percentage of beta-III-tubulin-positive neurons was performed using Arrayscan XTI HCA high-content imaging instrumentation (Thermo).

### Availability of supporting data

The data sets supporting the results of this article are available in the European Nucleotide Archive, IDs HG530137 and HG530138, http://www.ebi.ac.uk/ena/, as well as in the Addgene plasmid repository, IDs 48705 and 48706, http://www.addgene.org/.

## Results

**Figure 1:**
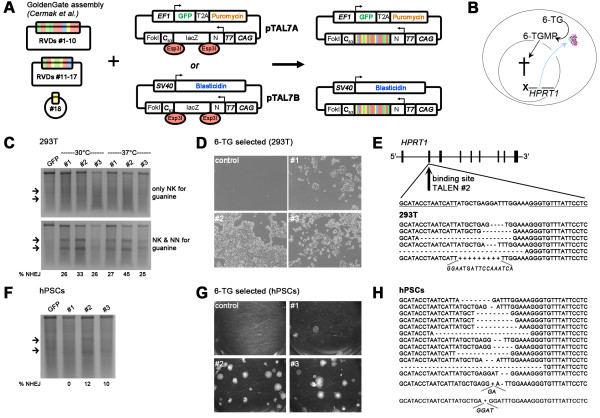
**Pretesting of TALENs and mutational spectra in 293 T cells and hPSCs. A**: Illustration of GoldenGate TALEN assembly by Cermak et al. [13] and cloning into new expression vectors pTAL7A and pTALB. Note that the number of RVDs is not limited to 18 but flexible. **B**: Schematic of negative selection of *HPRT1* knock-out cells by 6-thioguanine (6-TG). 6-TG results in death of cells with functional *HPRT1*. **C**: Cel-1 assay following transfection with three TALEN pairs (#1 to #3). Arrows indicate expected fragment sizes following Cel-1 digest of reannealed *HPRT1* genomic PCRs (gPCR) as a result of error-prone NHEJ. Control plasmid (GFP) transfected cells served as negative control. Percentage of non-homologous end-joining (NHEJ) is indicated below each lane. **D**: Surviving 293 T cells following TALEN transfection and 6-TG selection of *HPRT1* mutants. **E**: TALEN-induced mutation spectrum. Underlined wild-type sequence indicates binding region of TALEN pair #2. Mutant sequences shown are individual gPCR clones from bulk 6-TG selected cultures following transfection of TALEN pair #2. **F**: Pretesting of TALEN pairs #1 to #3 in HuES6 hESCs. Note that mutation efficiencies are somewhat decreased compared to 293 T cells in part B. **G**: Bulk hESC cultures following TALEN #2 transfection and 6-TG selection. **H**: TALEN-induced mutation spectrum in hESCs. All transfected cells were enriched by transient puromycin/blasticidin administration.

In order to create a vector system compatible with the GoldenGate transcription-activator like effector nuclease (TALEN) assembly kit [13] and to enable efficient expression in mammalian cells, two additional expression plasmids – pTAL7A and pTAL7B – were generated. The main features of these vectors include (i) a CAG promoter with a chimeric intron and a Kozak sequence for efficient TALEN expression in mammalian cells, (ii) Esp3I (BsmBI) restriction sites for GoldenGate cloning, (iii) a lacZ fragment for blue/white-screening in *E.coli*, (iv) an improved, truncated TALE backbone architecture similar to the one established by Miller *et al*. [6], as well as (v) individual selection cassettes for enrichment of double-transfected cells. Hence, pTAL7A contains a constitutively expressed Puromycin resistance gene linked to green fluorescent protein, whereas pTAL7B contains a Blasticidin resistance cassette (Table 1, Figure 1A, Additional file 1: Figure S1). This is to enrich for double-transfected cells in hard-to-manipulate cell lines such as human pluripotent stem cells (hPSCs) – embryonic stem cells (hESCs) and induced pluripotent stem cells (hiPSCs) – employing transient administration of the two antibiotics following TALEN transfection.

**Table 1:** Comparison of pTAL4 and pTAL7 vector systems

	**pTAL4**	**pTAL7**
Site for GoldenGate TALEN Assembly	Esp3I	Esp3I
Bacterial Resistance	Ampicillin	Kanamycin
Screening for successful cloning	LacZ	LacZ
TALEN architecture	Full length Voytas et al. [13]	Truncated C- & N-termini similar to Miller et al. [6]
FokI nuclease	Wild-type	Wild-type
Expression system	Yeast	Mammalian cells (CAG promoter + chimeric intron + Kozak sequence)
Enrichment of transfected cells	-	GFP & Puromycin (pTAL7A) Blasticidin (pTAL7B)
In-vitro transcription	-	T7 promoter
Codon usage	Not optimized for mammalian cells	Optimized for mammalian cells
Tag	-	Flag tag

To demonstrate the functionality of the combined system, we designed 3 pairs of TALENs targeting exon 2 of the *HPRT1* gene (Additional file 1: Table S1). *HPRT1* is located on the X chromosome and mutations in this gene cause Lesch-Nyhan-Syndrome (LNS), a disease with strong neurological symptoms [14,15]. Cells without functional *HPRT1* can be selected via 6-thioguanine, a guanine analogue that is metabolized by *HPRT1* and introduced into the DNA, resulting in mutagenesis and cell death (Figure 1B). Robust expression of cloned TALEN and selection cassettes in mammalian cells was confirmed by qRT-PCR (Additional file 1: Figure S2A,B). TALEN constructs were once more transfected into 293 T cells, transiently incubated at 37°C and 30°C to also investigate effects of low-temperature incubation on non-homologous end joining (NHEJ)-based mutation frequencies [6]. *HPRT1* PCRs on isolated genomic DNA were denatured, reannealed, and subjected to Cel-1 digestion and gel electrophoresis to reveal the generation of small genetic lesions in these bulk cultures. Specificity of DNA-binding of TALENs is mediated by two amino acids in each of the individual repeat domains, the so-called repeat variable di-residue (RVD). Several RVDs have been found to bind with different affinities to their target nucleotide [5]. Using TALENs employing only the “NK” RVD for targeting guanine did not produce detectable Cel-1 digestion fragments (Figure 1B, top panel). However, replacing “NK” by “NN” RVDs at strategic positions, as previously suggested [4,5], revealed that all three tested TALENs were functional as their delivery into 293 T cells apparently caused robust introduction of small lesions in *HPRT1* (Figure 1C, bottom panel, Additional file 1: Table S1). A transient cold-shock at 30°C did not have a significant effect on induction of double-strand breaks (DSBs) (Figure 1C). Functional selection of *HPRT1* mutant cells using the 6-TG confirmed these results in that all three TALENs produced 6-TG resistant cells, at varying efficiencies (Figure 1D). Sequencing of PCR clones from these 6-TG selected cultures showed that TALEN delivery mostly resulted in small deletions, implying that resulting frame shifts were the causes of disrupting HPRT1 function (Figure 1E).

We then asked if it was possible to pre-test TALEN pairs directly in hESCs. Indeed, the results were similar in that TALEN pair #2 appeared to cut its target site most efficiently, albeit the obtained Cel-1 signals were somewhat weaker than in 293 T cells (Figure 1F). 6-TG selection functionally confirmed the disruption of the *HPRT1* gene in hESCs as numerous resistant colonies appeared with two out of three TALEN pairs tested (Figure 1G). Analysis of the mutational spectrum in hESCs by sequencing of PCR clones revealed that mostly deletions as well as few insertions had been introduced by TALEN delivery, like in 293 T cells (Figure 1H).

**Figure 2:**
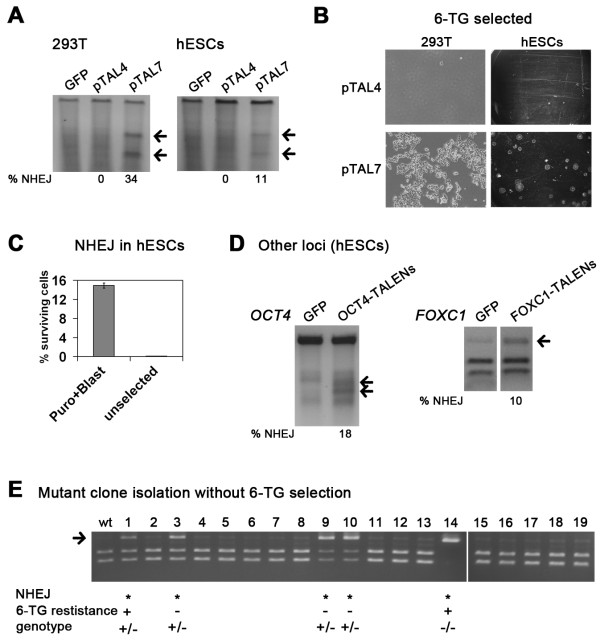
**Improved mutagenesis and universal applicability of the pTAL7 vector system. A**: Cel-1 assay following transfection of 293 T and hESCs with *HPRT1* talen pair #2 in different expression vector backbones. **B**: Functional confirmation of results in (A) by 6-TG selection. **C**: Quantification of functional mutations in *HPRT1* following 6-TG selection, based on scoring of colony numbers. For the control without antibiotics and the pTAL4 vectors, no puromycin/blasticidin selection was performed. Note the strong increase in mutation rates upon pre-selection for double-transfected cells using transient administration of antibiotics. Error bars: SEM (n = 4). **D**: Applicability of the pTAL7 system to alternative genomic loci as shown by Cel-1 assay (left panel) or restriction digestion (right panel: NHEJ is indicated by enrichment of undigested fragments). hESCs were transfected with either a GFP control vector or the respective TALEN vectors, followed by subsequent gPCR amplification of TALEN target regions. **E**: Restriction digestion and characterization of 19 clones expanded after TALEN transfection without 6-TG selection. Except in panel C, pTAL7-transfected cells were routinely enriched by transient puromycin/blasticidin administration.

To ask if the pTAL7 vectors indeed present an improvement over the existing platform using mammalian cells, the same TALEN pair #2 was employed both in the context of the original pTAL4 and the new pTAL7 expression vectors. Both in 293 T cells and hESCs, we failed to detect any evidence for error-prone NHEJ with the pTAL4 vectors (Figure 2A). Selection with 6-TG functionally confirmed these results (Figure 2B, Additional file 1: Figure S2C,D). Furthermore, we asked if the performance of our expression vectors could be further improved by replacing wild-type FokI nuclease domain by the *sharkey* variant which was previously reported to increase DSB formation [20]. However, with the present system, *sharkey* FokI did not yield higher numbers of 6-TG resistant cells (Additional file 1: Figure S2C,D). Despite this fact, these data suggest that the pTAL7 vectors enable robust targeted mutagenesis compared to the original system.

Next, we sought to quantify mutation frequencies in hESCs by determining the ratio of surviving 6-TG resistant hESC colony numbers by the total number of colonies emerging in the absence of 6-TG. Using pre-selection of double-transfected hESCs by puromycin and blasticidin, functional *HPRT1* mutations were introduced in approximately 15% of cells. Mutation rates without pre-selection were low (0.5%), reflecting the usefulness of these resistance cassettes in the pTAL7 vectors (Figure 2C).

In order to test the applicability of our system to loci other than *HPRT1*, we employed TALENs targeting the *OCT4*[1] as well as the *FOXC1* locus. Transfection into hESCs and subsequent analysis showed robust induction of error-prone NHEJ in both these genes (Figure 2D). Moreover, to demonstrate that clonal knock-out hESC lines could be isolated without the option of functional negative selection (as with 6-TG), randomly picked colonies emerging after *HPRT1* TALEN transfection were expanded and screened for genomic lesions. Mutant clone screening was in this case performed based on potential disruption of a restriction enzyme recognition site within the TALEN targeting region. Of the 19 clones analyzed, 5 showed an undigested PCR band (Figure 2E) suggesting at least heterozygous mutations. We further analyzed these clones using 6-TG as well as sequencing of the *HPRT1* locus. One clone showed small deletions in both alleles and was subsequently functionally confirmed to be 6-TG resistant (#14 in Figure 2E). These data demonstrate the universal applicability of the combined GoldenGate/pTAL7 TALEN system for generating knock-out hESC lines without the need for additional gene targeting vectors or negative selection procedures.

**Figure 3:**
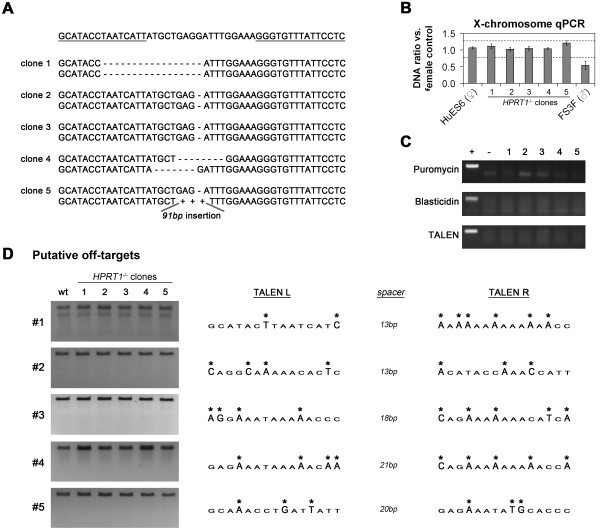
**Functional characterization of *HPRT1 *knock-out cell lines. A**: *HPRT1* lesions identified in 5 clonal hESC mutant lines. 10 PCR clones were sequenced per cell line. Wild-type sequences shown on top were not seen in any case suggesting that cell clones 1–3 are homozygous. Inserted DNA sequence of clone 5 is given in Additional file 1. **B**: Quantification of X chromosome copy number in clonal *HPRT1* mutant lines using genomic qPCR for an X-linked locus, based on qPCR quantification. Note that all mutant clones cluster with the female parental control. **C**: Diagnostic PCR for detecting possible random integration of vector sequences into the genome of TALEN-treated cell clones. Analysis was performed at passage 5 after 6-TG selection. Original pTAL7 vectors served as positive control and isogenic parental cells served as negative control. **D**: PCR and subsequent Cel-1 assay for detection of mutations at the 5 most probably putative TALEN off-target cleavage sites in clonal *HPRT1* knock-out cell lines. Genomic DNA from parental hESCs served as control. Note that the additional band in off-target site 1 probably results from a single nucleotide polymorphism and that there are no differences between clonal *HPRT1* knock-out cell lines and the parental control. TALEN off-target binding sites are depicted in the right panel with mismatches indicated by asterisks.

We next sought to demonstrate the usefulness of the system in the context of human disease modeling. To this end, 5 additional 6-TG resistant HuES6 hESC clones were expanded and sequenced. All 5 clones had mutations in both *HPRT1* alleles, with 3 clones being homozygous and 2 heterozygous (Figure 3A), suggesting a loss of X chromosome inactivation in all 5 clones [18]. Conversely, performing the same experiment with low-passage female hiPSCs that presumably have not undergone loss of X inactivation [18] produced 6-TG resistant clones with both homo- and heterozygous *HPRT* lesions (data not shown). To rule out that loss of one X-chromosome was the reason for detecting only one specific mutation in three of the hESC mutant clones, qPCR using an X-linked marker verified that all clones had retained both X-chromosomes (Figure 3B). Furthermore, no traces of the vector sequences were detectable by genomic PCR in these clones (Figure 3C), suggesting that transient antibiotic selection did not favor integration of the pTAL7 vectors. In order to investigate putative off-target cleavage of *HPRT1* TALENs, five putative off-target loci were screened for unwanted mutation events in the five clonal HPRT1 knock-out cell lines. Off-target effects were detected in none of these sites (Figure 3D).

**Figure 4:**
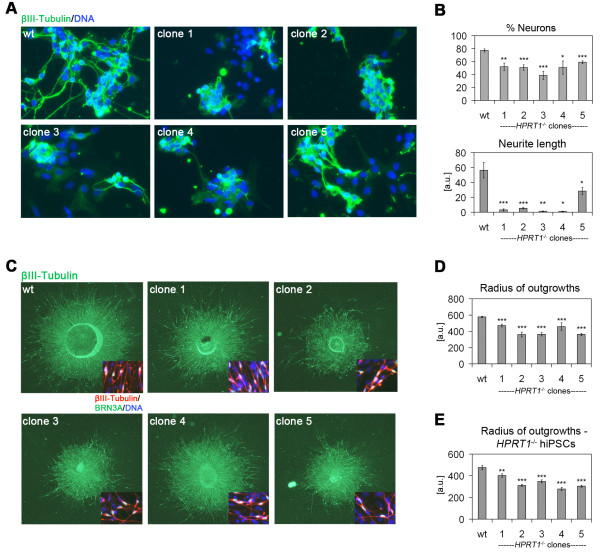
**Neural differentiation of HPRT1 knock-out cell lines. A**: Neuronal phenotypes in isogenic *HPRT1* mutant hESC lines. Beta-III-tubulin-stained CNS neurons derived from 5 independent *HPRT1* mutant lines and isogenic wild-type control. Note the higher numbers with more pronounced neurites in wild-type compared to mutant cells. **B**: High-content imaging-based quantification of neuron formation frequencies and neurite lengths of cultures shown in A. P-values are based on unpaired t-tests between individual clones and wild-type control samples. *: p ≤ 0.05; **: p ≤ 0.01; ***p ≤ 0.001. **C**: Neurons growing out in a radial manner from plated embryoid bodies induced to differentiate into BRN3A-positive PNS neurons. Images show representative outgrowths stained for beta-III-tubulin under 50x magnification. Sphere radius values served as an approximation for quantifying relative neurite lengths. **D**: Quantification of sizes of replicate plated spheres shown in C (n ≥ 7). **E**: Quantification of sizes of replicate plated spheres from *HPRT1* knock-out hiPSCs differentiated into sensory-like neurons as in C (n ≥ 8). P-values are based on unpaired t-tests between individual clones and wild-type controls. **: p ≤ 0.01; ***p ≤ 0.001.

In order to investigate whether neurons derived from *HPRT1* mutant cell lines recapitulated disease phenotypes *in vitro*, as recently shown in case of patient-specific Lesch-Nyhan Syndrome (LNS) hiPSCs [18], wild-type and mutant cell lines were differentiated into central nervous system (CNS) neurons. Compared to isogenic parental control cells, all 5 knock-out clones appeared to show impaired neuronal differentiation, based on immunofluorescence analysis (Figure 4A). Quantitative analysis of these differentiated cultures revealed a reduced overall number of neurons together with a strong decrease in average neurite length (Figure 4B). To see if a phenotype could also be assigned to neurons of the peripheral nervous system, we also conducted differentiation into sensory-like neurons [19]. Indeed, quantification of the average neurite length of BRN3A-postive neurons growing out from plated embryoid bodies revealed a similar phenotype with significantly shorter neurites in *HPRT1*-targeted cells (Figure 4C,D). To investigate if this relatively mild phenotype in sensory-like neurons could also be observed with independent cell lines, we generated *HPRT1* knock-out clones from induced pluripotent stem cells, in the same manner as described above for hESCs. Parental hiPSCs and five independent mutant clones were once more differentiated into BRN3A-positive neurons, to quantify resulting average neurite lengths. Again, loss of functional *HPRT1* resulted in significantly shorter neurites (Figure 4E) in the mutant clones as compared to parental controls. Taken together, these data demonstrate the applicability of the combined TALEN system to genetic disease modeling based on isogenic pairs of mutant and wild-type hPSC lines.

## Discussion

TALENs have become a valuable tool for genome editing in a variety of cell types, including hPSCs [1,7,21]. Over the past few years, various methods to assemble TALENs have been developed, ranging from gene synthesis to manual cloning kits and automated high-throughput systems [7-11,13]. The GoldenGate TALEN assembly kit by Cermak *et al.* is publicly available, straightforward to establish in the laboratory, reliable, as well as time and cost-efficient [13]. However, the final expression vectors of this kit were not optimized for application in mammalian cells. Furthermore, modifications of the TALEN domain architecture shown to improve DSB induction [6] were not included in the expression vectors. The pTAL7 vectors described here are fully compatible with the GoldenGate TALEN kit [13], yet overcome these drawbacks. They enabled robust induction of DSBs in human cell lines, including hPSCs, resulting in functional gene knock-out without the need of conventional targeting vectors. The selection cassettes implemented in the plasmids enable enrichment of double-transfected cells, which proved to be key for obtaining high mutation frequencies in our hands. Pre-selection for double transfectants will particularly be necessary in cases where negative functional selection as in case with *HPRT1*/6-TG is not an option. Indeed, we demonstrate that the pTAL7 vectors permitted the isolation of hPSC knock-out lines without applying 6-TG selection, based solely on random picking of clones. In comparison with enrichment methods relying on fluorescent marker proteins [7], the pTAL7 system offers both antibiotic and fluorescent selection of transfected cells, making it highly versatile and independent of the availability of cell sorting instrumentation. Furthermore, conventional lipofection as a delivery method yielded sufficient numbers of clones with small amounts of starting cells, in contrast to methods based on electroporation [1,7].

The disease phenotypes observed in *HPRT1* knock-out cell-derived CNS neurons recapitulated aspects of impaired neurogenesis in LNS patients and were in line with observations made with patient-specific hiPSCs, albeit showing an overall higher number of differentiated neurons in our hands [18]. These differences may be explained with differences among individual hPSC lines, supporting the necessity of isogenic disease models. Future experiments could address the effects of *HPRT1* knock-out on neuronal differentiation side-by-side in engineered hESCs and patent-specific hiPSCs. In addition, impaired neurite outgrowth was also observed in BRN3A-positive neurons [19], extending these findings to the PNS system. Notably, these phenotypes were observed in independent clones of independent cell lines (hESCs & hiPSCs) and in comparison to isogenic parental controls, which demonstrates a causative role of mutant *HPRT1* irrespective of the genetic background. It would be worthwhile further investigating this effect on sensory-like neurons to study functional links to LNS phenotypes. Taken together, the observed cellular phenotypes confirm that TALEN-mediated mutagenesis in wild-type hPSCs is a valid alternative for disease modeling without the need for patient material and lengthy reprogramming as well as hiPSC characterization procedures [22]. Introduction of more subtle lesions or gene correction approaches would be enabled by employing conventional gene targeting vectors in addition to the TALEN constructs [23]. Furthermore, the system is not limited to using hPSCs, as we have also successfully used it in HEK-293 T, HeLa, and mouse ES cells (Figure 1C-E, and data not shown).

The field of targeted genetic engineering is rapidly evolving. For example, RNA-protein-mediated DSB induction by the CRISPR-Cas9 system has recently been shown to efficiently enhance gene targeting in a variety of organisms and cell types, including hPSCs, similar to TALENs [22,24-30]. However, comprehensive studies addressing target specificity of this new platform are yet to be carried out, with regards to the shorter binding sequence of CRISPRs compared to TALENs as well as regarding the tolerance of single-base mismatches in the recognition sequence. Likely, future investigation will be based on several well-working genetic engineering systems that may be selectively employed depending on their respective strengths and weaknesses.

## Conclusion

The improved TALEN system evaluated in this work presents an affordable and easy-to-adopt methodology to facilitate gene targeting in human pluripotent stem cells and other mammalian cell types. It will thus be helpful for developmental studies as well as disease modeling approaches.

## Competing interests

The authors indicate no potential competing interests.

## Authors’ contributions

Designed and assembled the vector system: SF, BVS. Conceived and designed the experiments: SF, BG. Performed the experiments: SF. Analyzed the data: SF, BG. Wrote the paper: SF, BG. All authors read and approved the final manuscript.

## Supplementary Material

Additional file 1 Supporting Information, including detailed description of methods, TALEN sequences as well as additional figures. Click here for file
