# Supplementary material for: A modified TALEN-based system for robust generation of knock-out human pluripotent stem cell lines and disease models
Source: BMC Genomics. 2013 Nov 9;14:773. doi: 10.1186/1471-2164-14-773 (PMC3840567; doi:10.1186/1471-2164-14-773)
Supplement: Additional file 1 — Supporting Information, including detailed description of methods, TALEN sequences as well as additional figures. [file 1471-2164-14-773-S1.doc]

**Supporting Information**

**Detailed materials & methods**

**Cell culture**

hPSCs were cultured on Matrigel®-coated dishes (BD) in either MEF-conditioned medium or defined FTDA medium [1-2]. FTDA contained: DMEM-F12 (Life Technologies), 1x Insulin-Transferrin-Selenite (BD), 1:100 (v/v) chemically defined lipids (Life Technologies), 0.1% human serum albumin (Biological Industries), 10 ng/ml FGF2 (Peprotech), 0.2 ng/ml TGFβ1 (eBioscience), 5 ng/ml Activin A (eBioscience), 50nM Dorsomorphin (Santa Cruz), and 1x Penicillin-Streptomycin-L-Glutamine (PAA). For splitting cells, hPSC colonies were cut into equally sized fragments using a sterile needle. After washing once with PBS, the cells were incubated with 2 mg/ml Dispase (Life Technologies) for 5-10 min at 37°C and subsequently washed three times with PBS. Cells were then scraped off with a sterile cell scraper and transferred to a 15ml conical tube. After brief centrifugation (5s at 200g), clumps were resuspended in medium supplemented with 10µM Y27632 (Abcam) and transferred to equilibrated medium in Matrigel®-coated culture dishes. For transfection and clonal selection, cells were dissociated using Accutase®, counted, and seeded out as single cells to be transfected on the following day.

HEK-293T cells were cultured on tissue culture dishes in DMEM supplemented with 10% fetal bovine serum, 1x non-essential amino acids, 2-mercaptoethanol, and 1x Penicillin-Streptomycin-L-Glutamine.

**Design and construction of the pTAL7 vectors**

A fragment containing the CAG promoter, a nuclear localization signal (NLS) and a FLAG-tag epitope was released from the *pCAG-TAL-linker-IX_Fokwt* plasmid by enzymatic digestion with Asp718 and AscI (1.8 kb). The N-terminal part was amplified via PCR from the *pCAG-TAL-linker-IX_Fokwt* plasmid using the primer pair “TALEN_Nterm” (Table S2). The 0.55kb PCR product was then digested with Asp718 and BsmBI restriction endonucleases. In order to enable blue/white-selection of successfully assembled TALEN constructs, a spacer fragment containing the lacZα portion of the β-galactosidase gene flanked by Esp3I restriction sites was released from the chemically synthesized TAL_ELD cassette by BsmBI digestion (0.4kb). The truncated C-terminal part of the TALE cassette, the wild-type FokI nuclease domain and the SV40 transcriptional termination region were amplified via PCR from the *pCAG-TAL-linker-IX_Fokwt* plasmid using primer pair “TALEN_Cterm” (Table S2). The PCR product (1.4kb) was then digested with NotI and BsmBI restriction endonucleases. As backbone, the *pZErO-2* plasmid (Invitrogen) was amplified by PCR using the primer pair “pZE_backbone” and subsequently digested with AscI and NotI restriction endonucleases.

All DNA fragments were ligated in a single reaction and the resulting vector was verified by DNA sequencing and termed *pTAL7*.

The additional selection and fluorescent marker cassettes were amplified via PCR from available vectors (introducing BspHI restriction sites), subcloned into TOPO vectors (Invitrogen) and released by restriction endonulease BspHI. After digestion of the *pTAL7* plasmid with BspHI, the linearized vector was ligated with either an *EF1α-green fluorescent protein (GFP)-T2K-Puromycin (pTAL7A)* cassette or a *SV40-Blasticidin (pTAL7B)* cassette.

All PCR reactions were performed using Phusion® Polymerase (NEB) to minimize amplification errors.

The plasmid *pCAG-TAL-linker-IX_Fokwt* containing the TALEN cassette based on the AvrBs3 sequence from *Xanthomonas* bacteria was kindly provided by Prof. Dr. R. Kühn (Helmholtz Zentrum München, German Research Center for Environmental Health).

For testing the sharkey FokI nuclease domain, a DNA fragment containing the modified FokI sequence [3] was synthesized (Eurofins MWG Operon) to replace the wt FokI sequence in the pTAL7 vectors using PmeI/MluI restriction enzymes.

**TALEN design and assembly**

All TALEN pairs were designed using either the ZiFit targeter (http://zifit.partners.org/ZiFiT/) or the TAL Effector Nucleotide Targeter 2.0 (https://tale-nt.cac.cornell.edu/node/add/talen). Only TALEN pairs with 14-18 RVDs each and a spacer length of 14-18 bp were considered and 3 pairs selected for assembly.

The assembly was performed as published [4], by following the protocol associated with the GoldenGate TALEN and TAL Effector Kit 1.0 (Addgene #1000000016), with the final vectors of the second reaction being *pTAL7A* and *pTAL7B* (for antibiotic selection, 50µg/ml Kanamycin was used). Primers “pTAL7_screening_F” and “–R” (Table S2) were used for colony PCR as well as sequencing of the TALEN constructs. Two versions of each TALEN pair were generated, one only containing the repeat variable di-residue (RVD) NK for guanine (except at the last position, due to technical constraints of the assembly kit), the other containing the RVDs NK and NN for guanine (NK was replaced by NN at strategic positions according to Streubel *et al.* [5]). The *OCT4* TALEN sequences were adopted from a published report [6]. For the *FOXC1* TALENs, NH from the GoldenGate TALEN kit 2.0 (Addgene #1000000024) was used for guanine. All RVD and target sequences are given in Table S1.

**TALEN testing & *HPRT1* knock-out generation**

75,000 HEK-293T cells or 185,000 hPSCs were seeded per well of a 6-well plate 24h before transfection and then transfected with 2 or 4 µg of an equimolar mixture of both TALEN plasmids, respectively, using FuGene 6 (Roche). 24h after transfection, cells were selected for 48h with 0.5µg/ml of puromycin and 5µg/ml of blasticidin. For 30°C experiments, cells were transiently incubated at 30°C for one day starting from 6h after transfection. Antibiotic selection was initiated with a 6h delay in this case. After selection, cells were dissociated and analyzed via flow cytometry for enrichment of GFP-positive cells. A fraction of cells was lysed over night at 55°C in lysis buffer (10mM Tris-Cl pH=7.3, 10mM EDTA, 10mM NaCl, 0.5% SDS, 20 µg/ml Proteinase K) and genomic DNA was purified by ethanol precipitation and subsequent dissolving in 10mM Tris-Cl (pH=8.5).

In order to determine frequencies of NHEJ, 100ng of genomic DNA were used as template for the first PCR reaction with the primers HPRT_F1 and R1 (Table S2). Amplification was performed for 20 cycles with OneTaq® Polymerase (NEB). 1 µl of the 1^st^ PCR was used as template for nested PCR using primers HPRT_F1 and R2 (33 cycles). PCR products were purified by NaOAc-EtOH precipitation and DNA pellets were resuspended in 10mM Tris-Cl (pH=8.5) / 15mM MgCl_2_ / 50mM KCl. NHEJ-frequencies were analyzed with the Cel-1 assay (Surveyor Nuclease S, Transgenomic, #706020) according to the manufacturer’s protocol, followed by agarose gel electrophoresis (2%) and quantification as previously reported [7], using the ImageJ software . Testing the *OCT4* & *FOXC1* TALENs was performed similarly with the appropriate primer pairs. However, for *FOXC1*, the PCR reaction was performed with Q5 Polymerase (NEB, # M0491S) as a nested PCR using 1x GC-Enhancer and the Cel-1assay was replaced by PstI (Thermo Scientific, # FD0614) digestion, resulting in an enrichment of undigested PCR product as a consequence of NHEJ. Randomly picked *HPRT1* TALEN-treated were analyzed by restriction digestion of the PCR product with Bpu10I (NEB # R0649S).

For negative selection of *HPRT1* knock-out cells, 6-Thioguanine (Sigma, #A4862) was applied for 4-8 days at a concentration of 30µM. The efficiency of TALEN-mediated mutagenesis in hPSCs was calculated by dividing the number of surviving clones by the number of initially seeded cells while correcting plating efficiency, which was determined by seeding 1,000 control cells in parallel without 6-TG selection and counting of the emerging colonies. The relative efficiency of knock-out generation in 293T cells was done by relating numbers of 6-TG resistant cells to the number of initially seeded cells.

**Expression analysis, X-chromosome qPCR, detection of plasmid integration**

Analysis of vector expression was performed by qRT-PCR as described [1]. Cells were lysed 24h after transfection.

To determine the number of X chromosomes in isolated knock-out clones, qPCR for an arbitrary X-linked locus (in *SOX3*) was performed on genomic DNA. Relative X chromosome copy numbers were calculated using ΔΔCt values normalized to autosomal control loci (in *FGF2* and *DPPA4*).

In order to determine random integration of the TALEN vectors into the genome, knock-out cells at passage number 5 after picking were lysed to isolate genomic DNA. *pTAL7A* and *–B* vectors were used as positive controls and genomic DNA from isogenic wild-type hPSCs served as a negative control for the PCR reaction.

Primers used for amplification can be found in Table S2.

**Analysis of putative TALEN off-target cleavage sites**

Possible TALEN off-target sites were chosen with the “Paired Target Finder” tool from TAL Effector Nucleotide Targeter 2.0 (https://tale-nt.cac.cornell.edu/node/add/talef-off-paired) with spacers from 12 to 21bp. Off-target sites were sorted according to the sum of the affinity scores for both TALENs and the top 5 sites were chosen for further investigation. Genomic DNA from individual hESC *HPRT1* knock-out clones and parental wild-type hESCs was used for PCR amplification. For subsequent Cel-1 assays, an equal amount of PCR amplicon from each clone was mixed with wild-type PCR. Primers used for amplification can be found in Table S2.

**Neuronal differentiation and quantification**

Differentiation into neurons was performed as previously described [8-9]. The original isogenic wild-type control cell line was processed in parallel to mutant lines.

For PNS-like neurons, embryoid bodies were plated on Matrigel® to allow radial outgrowth of emerging neurons. 4 days after plating, cells were processed for immunocytochemistry. Neurite length was quantified by measuring the radius of the circular outgrowths of ≥7 spheres per clone.

CNS neurospheres were dissociated after 35 days of differentiation and seeded in different densities on Matrigel®. After 3 days of culture, cells were fixed and stained. Quantification of the percentage of βIII-Tubulin positive neurons and average neurite lengths was performed in an automated manner using Arrayscan XTI HCA high-content imaging instrumentation (Thermo) and in-house established analysis protocols.

**Immunocytochemistry**

For immunofluorescene analysis, cells were fixed for 10 min in 4% paraformaldehyde, permeabilized for 10 min in 1% Triton-X-100, blocked for 60 min in blocking solution (2% BSA/5% FCS, 2% glycine in PBS-T) at room temperature, and incubated with primary antibodies over night at 4°C in PBS-T with 0.5% BSA. Incubation with secondary antibody was performed a room temperature for 60 min in PBS-T with 0.5% BSA. After each step, cells were washed three times in PBS-T.

Nuclei were visualized by Hoechst staining. Primary antibodies used were: α-βIII-Tubulin (Covance, #PRB-435P, 1:2000; Sigma, #T8660, 1:1000; Covance, #MMS-435P, 1:500) and α-BRN3A (Santa Cruz, #sc-8429, 1:500). Secondary antibodies used were: goat α-mouse Alexa 488 (Invitrogen, #A11001, 1:1000) and goat α-rabbit Alexa 568 (Invitrogen, #11011, 1:1000).

**DNA sequences**

**Insertion in *HPRT1*-/- clone #5**

Tgtgaatttctttcttgttaatcaggaagaagatggtgggtaattaaacggctaattcgatagtgtgaaaggaacaacattattgccctgt

**Plasmid information**

Addgene Accession Numbers - pTAL7A: 48705; pTAL7B: 48706.

**Supplemental references**

1. Frank S, Zhang M, Schöler HR, Greber B: **Small molecule-assisted, line-independent maintenance of human pluripotent stem cells in defined conditions.** *PLoS One* 2012, **7:**e41958.

2. Xu C, Inokuma MS, Denham J, Golds K, Kundu P, Gold JD, Carpenter MK: **Feeder-free growth of undifferentiated human embryonic stem cells.** *Nat Biotechnol* 2001, **19:**971-974.

3. Guo J, Gaj T, Barbas CF, 3rd: **Directed evolution of an enhanced and highly efficient FokI cleavage domain for zinc finger nucleases.** *J Mol Biol* 2010, **400:**96-107.

4. Cermak T, Doyle EL, Christian M, Wang L, Zhang Y, Schmidt C, Baller JA, Somia NV, Bogdanove AJ, Voytas DF: **Efficient design and assembly of custom TALEN and other TAL effector-based constructs for DNA targeting.** *Nucleic Acids Res* 2011, **39:**e82.

5. Streubel J, Blucher C, Landgraf A, Boch J: **TAL effector RVD specificities and efficiencies.** *Nat Biotechnol* 2012, **30:**593-595.

6. Hockemeyer D, Wang H, Kiani S, Lai CS, Gao Q, Cassady JP, Cost GJ, Zhang L, Santiago Y, Miller JC, et al: **Genetic engineering of human pluripotent cells using TALE nucleases.** *Nat Biotechnol* 2011, **29:**731-734.

7. Guschin DY, Waite AJ, Katibah GE, Miller JC, Holmes MC, Rebar EJ: **A rapid and general assay for monitoring endogenous gene modification.** *Methods Mol Biol* 2010, **649:**247-256.

8. Mekhoubad S, Bock C, de Boer AS, Kiskinis E, Meissner A, Eggan K: **Erosion of dosage compensation impacts human iPSC disease modeling.** *Cell Stem Cell* 2012, **10:**595-609.

9. Greber B, Coulon P, Zhang M, Moritz S, Frank S, Muller-Molina AJ, Arauzo-Bravo MJ, Han DW, Pape HC, Scholer HR: **FGF signalling inhibits neural induction in human embryonic stem cells.** *EMBO J* 2011, **30:**4874-4884.

**Supplemental figures**

**Figure S1.** Vector maps of pTAL7 plasmids.


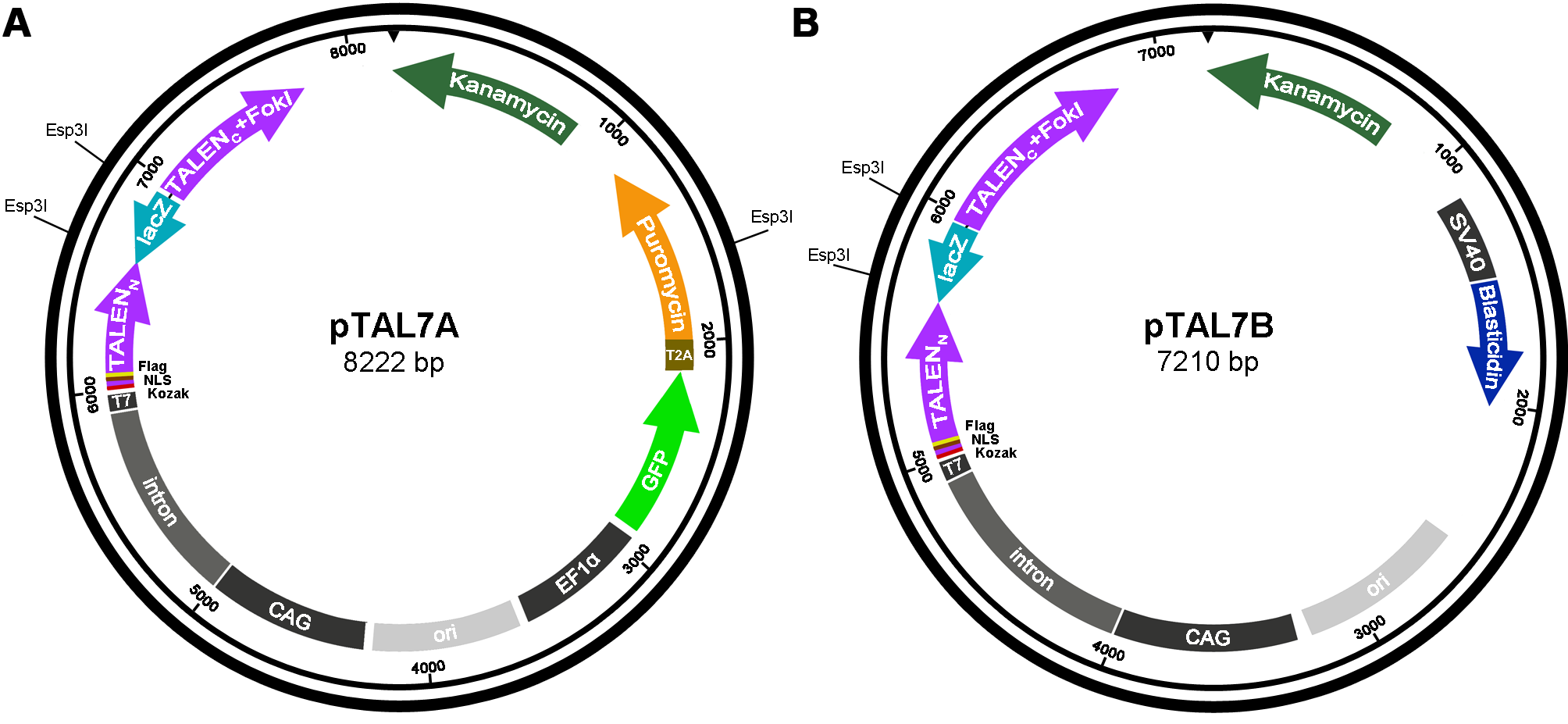


(A): pTAL7A carrying an indicated GFP-Puromycin cassette. (B): pTAL7B carrying a Blasticidin cassette. The pCAG-driven TALEN cassettes in both vectors contain a chimeric intron and a Kozak sequence for efficient expression in mammalian cells, as well as a Flag tag and nuclear localization signal (NLS). The additional Esp3I site in the Puromycin cassette of pTALA was confirmed to not interfere with the GoldenGate assembly.

**Figure S2.** Analysis of mRNA expression and functional validation of pTAL7 vectors.


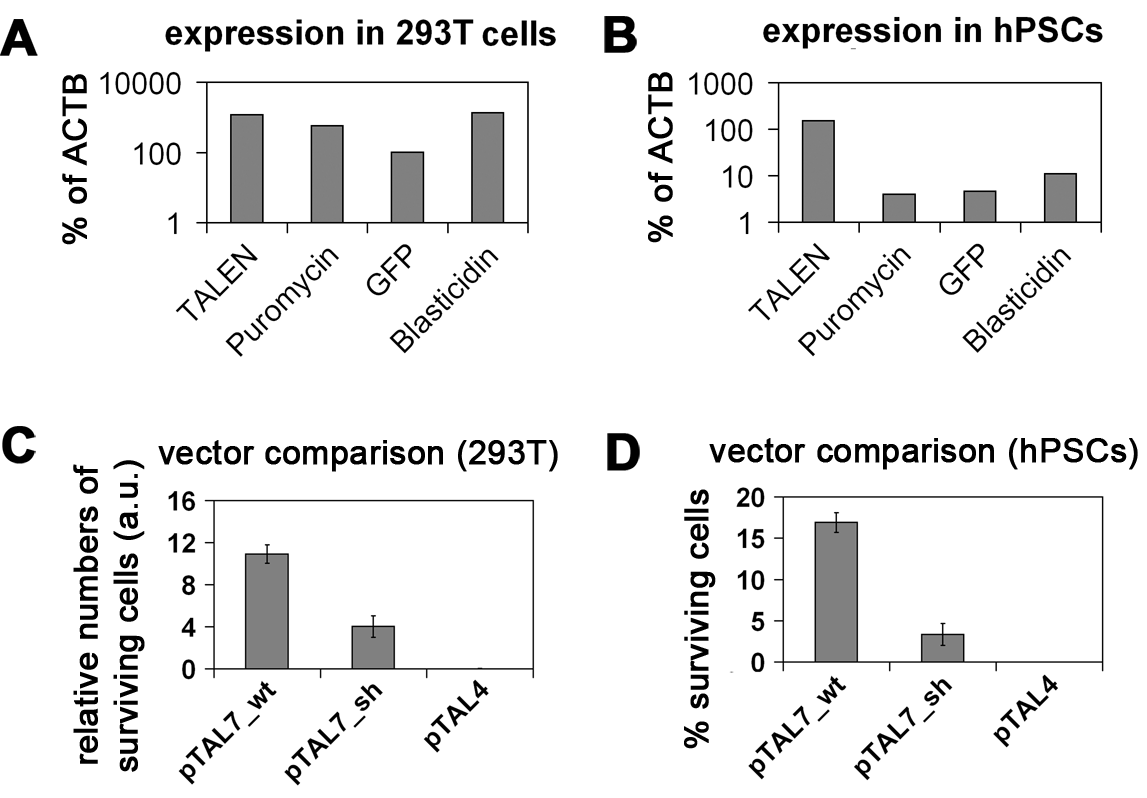


(A,B): Relative mRNA expression from vectors pTAL7A and –B in HEK-293T (A) and hPSCs (B), expressed as percentage of housekeeping ACTB levels. No amplification was detected in untransfected control cells. (C,D): Comparison of the pTAL7 vector system with a modified version carrying the *sharkey* FokI domain as well as with the original pTAL4 vector from the GoldenGate TALE assembly kit. *HPRT1* TALEN pair#2 was used in all cases. Error bars represent SEM (n=4).

**Supplemental tables**

**Table S1.** DNA target sequences and repeat variable di-residue (RVD) sequences of TALENs.

| TALEN pair # | TALEN A | TALEN B |
| --- | --- | --- |
| *1 (HPRT1)* | *TTTGCATACCTAATCATT* | *GAGGAATAAACACCCTT* |
| 1 (NK) | NG NG NG NK HD NI NG NI HD HD NG NI NI NG HD NI NG NG | NK NI NK NK NI NI NG NI NI NI HD NI HD HD HD NG NG |
| 1 (NN) | NG NG NG NK HD NI NG NI HD HD NG NI NI NG HD NI NG NG | **NN** NI NK **NN** NI NI NG NI NI NI HD NI HD HD HD NG NG |
| *2 (HPRT1)* | *GCATACCTAATCATT* | *GAGGAATAAACACCC* |
| 2 (NK) | NK HD NI NG NI HD HD NG NI NI NG HD NI NG NG | NK NI NK NK NI NI NG NI NI NI HD NI HD HD HD |
| 2 (NN) | NK HD NI NG NI HD HD NG NI NI NG HD NI NG NG | **NN** NI NK **NN** NI NI NG NI NI NI HD NI HD HD HD |
| *3 (HPRT1)* | *AATCATTATGCTGAG* | *TAGTCCATGAGGAAT* |
| 3 (NK) | NI NI NG HD NI NG NG NI NG NK HD NG NK NI NN | NG NI NK NG HD HD NI NG NK NI NK NK NI NI NG |
| 3 (NN) | NI NI NG HD NI NG NG NI NG **NN** HD NG NN NI NN | NG NI **NN** NG HD HD NI NG **NN** NI NK **NN** NI NI NG |
| OCT4 | *CACCTGCAGCTGCCCAG* | *GACCCTGCCTGCTCCT* |
| OCT4 | HD NI HD HD NG NN HD NI NN HD NG NN HD HD HD NI NN | NN NI HD HD HD NG NN HD HD NG NN HD NG HD HD NG |
| FOXC1 | *GAGCGTGTACTCGCAC* | *GCCGCCCGGGTACTGC* |
| FOXC1 | NH NI NH HD NH NG NH NG NI HD NG HD NH HD NI HD | NH HD HD NH HD HD HD NH NH NH NG NI HD NG NH HD |

DNA target sequences depicted in italics (5'→3'). RVD sequences are shown for two variants of each TALEN pair, the first with only NK targeting guanine and the second with NK and NN for guanine. NK was replaced by NN at strategic positions (bold, underlined) according to Streubel *et al.* [5]. Note that for TALEN 3A the last RVD is NN in both cases as predefined by the GoldenGate TALEN assembly kit.

**Table S2.** Primer Sequences (5'→3').

| Primer pair | Forward | Reverse |
| --- | --- | --- |
| TALEN_Nterm | GGCCGCCACCATGGGACCTAAG | CCTGTAGCGGCGCCCGTCTCCAGGGGGGCTCCTGTCAGAGCATT |
| TALEN_Cterm | GCTATTACGCCAGCTGGCGAAAGGGGGATGTG | CCGCTTTCCACCGGTCGTCTCCAACGATCATCTGGTCGCTCTC |
| pZE_backbone | TTTCCACACCGGCGCGCCCAGGGGATAACGCAGGAAAGAAC | TTTTTGTGTGGCGGCCGCTTCAGAAGAACTCGTCAAGAAGG |
| pTAL7_screening | CGCTTGGAGGAATGCTCTGA | AAGCGAGAGCGACCAGATGAT |
| TALEN_qPCR | TGGAGGAAAACCAGACAAGAA | GGCCTGACACGAAGAGAAAC |
| Puro_ qPCR | CACCAGGGCAAGGGTCTG | GCTCGTAGAAGGGGAGGTTG |
| GFP_ qPCR | TGATGGGCTACGGCTTCTAC | GTACTTCTCGATGCGGGTGT |
| Blast_ qPCR | ATGAGAACAGGGGCATCTTG | GGCTGTCCATCACTGTCCTT |
| HPRT_exon2 | CCGGCCTGTTGTTTTCTTAC | R1 : GCCAGACATACAATGCAAGC  R2 : CCCCAGTCCATTACCCTGTT |
| SOX3 | GCAGTTAGCCAAGGAGTGAATGG | GGCGTAGGCGTTGCAGTTCT |
| FGF2 | GGCAAGATGCAGGAGAGAGGA | GCCACGTGAGAGCAGAGCAT |
| DPPA4 | TGGTGTCAGGTGGTGTGTGG | CCAGGCTTGACCAGCATGAA |
| OCT4 [6] | TAGGAGATGTGAGAGACCCTGACAAGG | AGGCGGCTTGGAGACCTCTCA |
| FOXC1 | F1 : CCGCCCTGGTTATTTGGCCG  F2 : CTACTCCGTGTCCAGCCCCAA | R1 : GGTGCAGCCTGTCCTTCTCCT  R2 : GCCCTGCTTGTTGTCCCGG |
| Off-target #1 | GACACCCATGCACAAGGACA | R1 : TGGTATCCAGCACAGCATCC  R2 : TCATTGACATGGAGCACAGTGGC |
| Off-target #2 | TTCATTGCCCTGACTCTTACCA | TCGCCAAGTTCCATTCTCAGA |
| Off-target #3 | GGGCCTAACACAACCCAGAT | AGCCCAAGAACGTGTAGCAA |
| Off-target #4 | AGCGATTATGTCAAGGGCCTG | R1 : GGCGAACCTCCATGTACCAT  R2 : GTGCATGCCTGTAGTCCCA |
| Off-target #5 | ACCTGGTGTGTATTCTAAGTCGAC | GGGCCCATTGTAGAAGAACCA |
